# Supplementary material for: A Glimpse into the Role and Effectiveness of Splenectomy for Isolated Metachronous Spleen Metastasis of Colorectal Cancer Origin: Long-Term Survivals Can Be Achieved
Source: J Clin Med. 2024 Apr 18;13(8):2362. doi: 10.3390/jcm13082362 (PMC11050850; doi:10.3390/jcm13082362)
Supplement: Supplementary file 1 [file jcm-13-02362-s001.zip › jcm-2927760-supplementary.pdf]

Journal of
Clinical Medicine

MDPI

Supplementary Table S1. Characteristics of 83 patients with splenectomies for metachronous isolated SM of CRC origin.

No	Year	Author, Reference	Sex	Age	Primary tumor	Interval (months)	Splenectomy	Number of SM	SM diameter, cm	Chemotherapy after SM resection	Survival (months)	Status
1	1965	Miller [25]	m	65	right colon	33	Open	1	5	NA	18	alive, no recurrence
2	1969	Dunbar [26]	m	69	rectum	48	Open	1	18	no	84	DOD
3	1982	Waller [27]	m	72	sigmoid	48	Open	1	9	NA	6	alive, with recurrence
4	1986	Slavin [28]	f	81	right colon	30	Open	1	NA	NA	12	alive, no recurrence
5	1992	Capizzi [29]	f	51	rectum	48	Open	1	3.5	no	14	alive, no recurrence
6	1993	Thomas [30]	f	72	sigmoid	132	Open	1	3	no	66	DOD
7	1995	Pedrazzoli [31]	m	65	left colon	18	Open	1	7	no	20	alive, no recurrence
8	1997	Mainprize [32]	f	62	left colon	42	Open	1	NA	NA	NA	NA
9	1997	Ishida [33]	f	73	colon (ns)	NA	Open	NA	NA	NA	72	alive, no reccurence
10			m	62	colon (ns)	NA	Open	NA	NA	NA	24	alive, no reccurence
11			m	52	colon (ns)	12	Open	NA	NA	NA	6	alive, no reccurence
12			m	48	colon (ns)	24	Open	NA	NA	NA	3	alive, no reccurence
13	1997	Indudhara [34]	m	74	sigmoid	24	Open	1	9.5	no	12	alive, no recurrence
14	1999	Weathers [35]	f	33	sigmoid	3	Open	1	3.5	no	12	alive, no recurrence
15	1999	Achuthan [36]	m	41	rectum	12	Open	NA	NA	no	NA	NA
16	2000	Kim [16]	m	65	right colon	37	Open	1	1	no	18	alive, no recurrence
17	2000	Fujita [17]	m	75	right colon	20	Open	1	9	yes (ns)	14	alive, no recurrence
18	2000	Lee [37]	f	66	colon (ns)	108	Open	1	NA	no	5	alive, no recurrence
19	2001	Place [38]	m	51	sigmoid	72	Open	1	13	no	6	alive, no recurrence
20	2001	Okuyama [18]	m	62	sigmoid	25	Open	1	3	no	20	alive, no recurrence
21		Japanese literature cases reported by Okuyama (1987 - 1999) [18]	m	64	right colon	3	Open	1	NA	NA	2	alive, no recurrence
22			f	61	right colon	26	Open	NA	NA	NA	15	DOD
23			m	56	transverse colon	6	Open	1	6	NA	14	DOD
24			m	74	left colon	48	Open	1	11.9	NA	NA	NA

25			m	55	transverse colon	42	Open	1	NA	NA	NA	NA
26			m	80	left colon	48	Open	1	4	NA	NA	NA
27			f	51	sigmoid	12	Open	1	9	NA	11	alive, no recurrence
28			m	65	rectum	39	Open	1	11	NA	41	alive, no reccurence
29			f	56	right colon	10	Open	1	2	NA	7	alive, no recurrence
30			m	74	left colon	18	Open	1	1.5	NA	12	alive, no recurrence
31			f	75	left + right colon	6	Open	2	3.6	NA	NA	NA
32			m	59	left colon	24	Open	1	4.5	NA	13	alive, no recurrence
33			m	58	right colon	11	Open	1	9	NA	12	alive, no recurrence
34			m	56	right colon	7	Open	1	4.5	NA	8	DOD
35			f	60	sigmoid	18	Open	1	NA	NA	NA	NA
36			f	82	sigmoid	24	Open	1	4	NA	11	DOD
37			f	56	right colon	8	Open	1	4	NA	13	DOD
38			f	51	sigmoid	48	Open	1	2	NA	16	alive, no recurrence
39	2003	Genna [39]	f	73	left colon	72	Open	1	6	NA	36	alive, no recurrence
40	2004	Hashemzadeh [40]	m	44	right colon	180	Open	1	14	NA	4	alive, no recurrence
41	2004	Pizzirusso [41]	m	66	left colon	5	Open	1	NA	yes (ns)	12	alive, no recurrence
42	2004	Cavallaro [42]	f	55	sigmoid	18	Open	1	3	NA	12	alive, no recurrence
43	2006	Lobato [43]	f	57	sigmoid	12	Lap	2	4	yes (ns)	12	alive, with recurrence
44	2006	Avninder [44]	f	52	sigmoid	108	Open	1	13	5FU	22	alive, no recurrence
45	2006	Gencosmanoglu [45]	m	76	left colon	17	Open	1	6.5	no	12	alive, no recurrence
46	2008	Popovic [46]	m	72	rectum	18	Open	1	12	NA	NA	NA
47	2008	Bigot [47]	f	69	left colon	24	Open	1	4	no	60	alive, no recurrence
48	2008	Gasent Blesa [48]	f	52	left colon	36	Lap	1	4.5	NA	NA	NA
49	2008	Montemurro [49]	f	80	right colon	31	Open	1	8	TT	6	alive, no recurrence
50	2009	Sileri [50]	m	73	right colon	63	Open	1	1.5	no	40	alive, no recurrence
51	2010	Busic [51]	m	72	left colon	24	Open	1	8	no	12	alive, no recurrence
52	2010	Genc [52]	m	59	right colon	15	Open	1	4	no	24	alive, no recurrence
53	2010	Dogan [53]	m	58	colon (ns)	20	Open	1	3.5	FOLFOX	18	alive, with recurrence

54	2011	El M'rabet [54]	m	46	right colon	60	Open	2	2.8	XELOX	36	alive, no recurrence
55	2011	Jain [55]	f	22	rectum	15	Lap	1	1	FOLFIRI, TT	58	alive, no recurrence
56	2011	Gatenby [56]	f	72	right colon	34	Open	NA	NA	FOLFOX, TT	58	alive, with recurrence
57			f	54	right colon	13	Open	NA	NA	5FU	16	DOD
58	2011	Pavlovic [57]	m	74	right colon	49	Open	1	7	no	6	alive, with recurrence
59	2012	Chekrine [58]	m	74	left colon	60	Open	1	5	no	7	alive, with recurrence
60	2013	Takeuchi [59]	m	68	right colon	36	Lap	1	2.5	yes (ns)	36	alive, no recurrence
61	2013	Lopez Monclova [60]	m	74	colon (ns)	NA	Lap	1	NA	NA	2	alive, no reccurence
62			f	60	colon (ns)	NA	Lap	1	NA	NA	2	alive, no reccurence
63	2014	Toyoshima [61]	m	79	sigmoid	19	Open	1	1.8	FOLFOX	12	alive, no recurrence
64	2016	Altaf [20]	m	53	sigmoid	12	Open	1	4.2	yes (ns)	24	DOD
65			m	59	sigmoid	3	Lap	1	NA	yes (ns)	12	alive, with recurrence
66	2016	Badak [62]	m	68	sigmoid	60	Open	1	3	no	6	alive, no recurrence
67	2016	Efared [63]	f	59	sigmoid	36	Open	2	6	yes (ns)	10	alive, no recurrence
68	2016	Tartaro [64]	f	84	right colon	7	Lap	1	8	NA	NA	NA
69	2016	Abdou [65]	f	64	right colon	16	Lap	1	4.9	5FU, TT	12	alive, no recurrence
70	2017	Lucke -Wold [66]	f	53	right colon	12	Open	1	3.5	no	36	alive, no recurrence
71	2017	Gilardi [67]	f	58	left colon	18	Lap	1	NA	NA	NA	NA
72	2017	Rizzo [68]	f	76	left colon	28	Open	1	1.6	capecitabi ne	21	alive, no recurrence
73	2019	Dimitrov [69]	f	43	rectum	14	Lap	1	NA	no	6	alive, no reccurence
74	2019	Kurumiya [21]	f	73	right colon	31	Open	1	3	no	87	alive, no recurrence
75	2019	Miller-Ocuin [70]	f	59	rectum	60	Lap	1	5.8	no	6	alive, with recurrence
76	2020	Hu [71]	f	48	sigmoid	21	Lap	mx	3	FOLFIRI, TT	7	alive, no recurrence
77	2021	Ognerubov [72]	f	68	left colon	21	Open	mx	2.5	XELOX	5	alive, no recurrence
78			f	70	left colon	10	Open	1	2.5	XELOX	26	alive, no recurrence
79	2022	Totikov [73]	f	62	sigmoid	28	Open	1	16	NA	NA	NA
80	2023	Xu [74]	m	41	rectum	60	Lap	1	4.2	FOLFIRI, TT	3	alive, no recurrence
81	2023	Chaudhary [75]	m	71	sigmoid	24	Lap	1	6.6	yes (ns)	1	alive, no recurrence
82	2023	Ramos [76]	f	41	right colon	24	Open	1	6	no	NA	NA

83	2024	Hong [77]	f	67	rectum	136	Open	1	8.4	no	6	alive, no recurrence

SM – splenic metastasis; CRC – colorectal cancer; Lap – laparoscopic; mx – multiple metastases; TT – targeted therapy; DOD – died of disease; NA – not available data; ns – not specified.
